# Supplementary material for: Complexome profiling of the Chlamydomonas psb28 mutant reveals TEF5 as an early PSII assembly factor
Source: Plant Cell. 2025 Mar 17;37(6):koaf055. doi: 10.1093/plcell/koaf055 (PMC12164590; doi:10.1093/plcell/koaf055)
Supplement: koaf055_Supplementary_Data [file koaf055_supplementary_data.zip › psb28_interactive.zip!/plots/intensity/qualityPlots/peptides/_Cre03.g195388.t1.1.html]

### Cre03.g195388.t1.1

Trivial name:   
SeqCoverage [median]: 18.40  
Mass: 9.29 kDa  
  
 

| Sequence | MissedCleavage | Mass [Da] | wtIntensity | muIntensity | Score |
| --- | --- | --- | --- | --- | --- |
| SELLQQQR | 0 | 1000.53 | 700.74 | 0.00 | 154.31 |
| TGEVKPSF | 1 | 863.44 | 776.42 | 0.00 | 94.11 |
|  |  |  | 1477.2 | 0.0 |  |
